# *Notes from the Field*: Online Weight Loss Supplements Labeled as Tejocote (*Crataegus mexicana*) Root, Substituted with Yellow Oleander (*Cascabela thevetia*) — United States, 2022

**DOI:** 10.15585/mmwr.mm7237a3

**Published:** 2023-09-15

**Authors:** Noah Berland, James Kababick, Cynthia Santos, Diane P. Calello

**Affiliations:** ^1^New Jersey Poison Information and Education System, Newark, New Jersey; ^2^Department of Emergency Medicine, Division of Medical Toxicology, Rutgers New Jersey Medical School, Newark, New Jersey; ^3^Flora Research Laboratories, Grants Pass, Oregon.

In the United States, dietary supplements are regulated by the Food and Drug Administration (FDA).* Regulations mandate that all ingredients used to manufacture dietary supplements be tested for identity and be free from reasonably anticipated contaminants. Despite these regulations, misbranded dietary supplements are frequently found to contain potentially dangerous substances (*1*). Tejocote (*Crataegus mexicana*) root, a supplement promoted online through social media for weight loss, is readily available from online retailers. Recent DNA fingerprinting of a product labeled as containing tejocote root under the brand name Alipotec determined that the product was 100% yellow oleander (*Cascabela thevetia*) (*2*). Yellow oleander contains the cardenolide thevetin B, which has the same clinical effects as other cardenolides, such as digoxin, and can be highly toxic.

## Investigation and Outcomes

On September 8, 2022, an emergency physician called the New Jersey Poison Information and Education System (NJPIES) regarding a child aged 23 months who had consumed Eva Nutrition Mexican Tejocote Root and developed nausea and vomiting. The product was marketed as a weight loss supplement and purchased by the patient’s mother. The patient was experiencing age-specific bradycardia (heart rate = 90 bpm; normal range = 98–135 bpm) and was hypotensive (blood pressure = 71/60 mm Hg). Electrocardiogram (ECG) results demonstrated sinus bradycardia, frequent premature ventricular complexes, and scooped ST segments consistent with cardenolide toxicity. At the direction of NJPIES, a serum digoxin assay was obtained with a reported level of 0.5 ng/L, which NJPIES interpreted as being attributable to cross-reactivity with a nondigoxin cardiac glycoside. After receiving treatment with 40 mg of digoxin-specific antibody fragments (FAB, a digoxin overdose antidote), the patient’s ECG and blood pressure normalized. A repeat ECG 12 hours later again demonstrated evidence of cardenolide toxicity. The patient received a second dose of FAB, and the ECG results returned to normal.

## Preliminary Conclusions and Actions

Because of the public health concerns of this likely mislabeled product, 10 products labeled as tejocote and marketed as weight loss supplements were purchased by NJPIES online during December 2022. Each product was listed on a separate page, although some carried the same or similar labels. Products were shipped directly to Flora Research Laboratories (Grants Pass, Oregon), which specializes in the analysis of chemical constituents found in dietary supplements. Using ultra-high pressure liquid chromatography–accurate mass-time of flight mass spectrometry analysis, researchers compared the purchased supplements with authenticated tejocote root procured and authenticated results with an ethnobotanist (Trish Flaster, Botanical Liaisons, personal communication, December 2022). Nine of 10 products labeled as tejocote were yellow oleander, with no evidence of tejocote root (Table).

**TABLE:** Results of testing* for presence of yellow oleander and tejocote root in 10 products labeled as containing tejocote — United States, January–May 2023

Product description	Yellow oleander detection	Tejocote root detection
Alipotec tejocote root pieces	Positive	Negative
Alipotec tejocote root pieces	Positive	Negative
Alipotec tejocote root capsules	Positive	Negative
Elv Alipotec Mexican tejocote root pieces	Positive	Negative
Eva Nutrition Mexican tejocote root pieces	Positive	Negative
Eva Nutrition Mexican tejocote root pieces	Positive	Negative
Niwali tejocote Mexican root pieces	Positive	Negative
Science Alpha Mexican tejocote root pieces	Positive	Negative
Tejocote seed liquid drops	Negative	Negative
Tejocotex tejocote root pieces	Positive	Negative

* Ultra-high pressure liquid chromatography–accurate mass-time of flight mass spectrometry analysis.

These readily available dietary supplements, upon testing, appeared to be mislabeled. Instead, they contained a toxic substance of concern to both clinicians and public health officials. FDA recently released a consumer warning about toxic yellow oleander purported to be Nuez de la India in certain botanical weight loss products.^†^ Clinicians need to be aware that persons with signs and symptoms of cardiac glycoside exposure might have been exposed to products labeled as tejocote, Nuez de la India, or other supplements marketed for weight loss and might benefit from treatment with a similar approach to that used in cases of nondigoxin cardiac glycoside exposures. Persons who are exposed to yellow oleander with evidence of toxicity might have a positive serum digoxin result on immune assays caused by cross-reactivity and might respond to FAB, as did the patient in this report. However, higher doses of FAB might be required for the reversal of yellow oleander toxicity than that typically used in cases of digoxin toxicity (*3*). Serum digoxin assays are not reliable for detection of thevetin B and cardiac glycosides other than digoxin. Laboratory-reported digoxin levels do not accurately reflect serum levels of other cardiac glycosides (*4*,*5*). For public health officials, this is concerning because these supplements contain a highly toxic substance and are readily available from multiple retailers. Future prevention efforts need to include reporting products such as these to FDA and alerting retailers who might be unknowingly selling these hazardous products. Clinicians will need to ask persons seeking care with evidence of cardiac glycoside toxicity about the use of weight loss supplements and consider FAB for treatment.

## Footnotes

*
https://www.ecfr.gov/current/title-21/chapter-I/subchapter-B/part-111
